# Supplementary material for: Resolving the full spectrum of human genome variation using Linked-Reads
Source: Genome Res. 2019 Apr;29(4):635–45. doi: 10.1101/gr.234443.118 (PMC6442396; doi:10.1101/gr.234443.118)
Supplement: Supplemental Material [file supp_gr.234443.118_Supplemental_longranger-2.2.zip › longranger-2.2.tar.bz2!/lr2.2-public/lib/stan/cmdstan/stan/doc/api/html/structstan_1_1gm_1_1statement__grammar.html]

Stan: stan::gm::statement\_grammar< Iterator > Struct Template Reference


|  |  |
| --- | --- |
|  | Stan  2.5.0  probability, sampling & optimization |


- Main Page
- Related Pages
- Namespaces
- Classes
- Files

- Class List
- Class Index
- Class Hierarchy
- Class Members

All Classes Namespaces Files Functions Variables Typedefs Enumerations Enumerator Friends Macros Pages

- stan
- gm
- statement\_grammar

Public Member Functions |
Public Attributes |
List of all members

stan::gm::statement\_grammar< Iterator > Struct Template Reference

`#include <statement_2_grammar.hpp>`

Inheritance diagram for stan::gm::statement\_grammar< Iterator >:

|  |  |
| --- | --- |
| Public Member Functions | |
|  | statement\_grammar (variable\_map &var\_map, std::stringstream &error\_msgs) |
|  | |

|  |  |
| --- | --- |
| Public Attributes | |
| variable\_map & | var\_map\_ |
|  | |
| std::stringstream & | error\_msgs\_ |
|  | |
| expression\_grammar< Iterator > | expression\_g |
|  | |
| var\_decls\_grammar< Iterator > | var\_decls\_g |
|  | |
| statement\_2\_grammar< Iterator > | statement\_2\_g |
|  | |
| boost::spirit::qi::rule  < Iterator, assignment(var\_origin),   whitespace\_grammar< Iterator > > | assignment\_r |
|  | |
| boost::spirit::qi::rule  < Iterator, expression(var\_origin),   whitespace\_grammar< Iterator > > | non\_lvalue\_assign\_r |
|  | |
| boost::spirit::qi::rule  < Iterator, std::vector  < expression >var\_origin),   whitespace\_grammar< Iterator > > | dims\_r |
|  | |
| boost::spirit::qi::rule  < Iterator, distribution(var\_origin),   whitespace\_grammar< Iterator > > | distribution\_r |
|  | |
| boost::spirit::qi::rule  < Iterator,   increment\_log\_prob\_statement(bool,   var\_origin),   whitespace\_grammar< Iterator > > | increment\_log\_prob\_statement\_r |
|  | |
| boost::spirit::qi::rule  < Iterator,   boost::spirit::qi::locals  < std::string >, for\_statement(bool,   var\_origin, bool),   whitespace\_grammar< Iterator > > | for\_statement\_r |
|  | |
| boost::spirit::qi::rule  < Iterator, while\_statement(bool,   var\_origin, bool),   whitespace\_grammar< Iterator > > | while\_statement\_r |
|  | |
| boost::spirit::qi::rule  < Iterator, print\_statement(var\_origin),   whitespace\_grammar< Iterator > > | print\_statement\_r |
|  | |
| boost::spirit::qi::rule  < Iterator, reject\_statement(var\_origin),   whitespace\_grammar< Iterator > > | reject\_statement\_r |
|  | |
| boost::spirit::qi::rule  < Iterator, return\_statement(var\_origin),   whitespace\_grammar< Iterator > > | return\_statement\_r |
|  | |
| boost::spirit::qi::rule  < Iterator, return\_statement(var\_origin),   whitespace\_grammar< Iterator > > | void\_return\_statement\_r |
|  | |
| boost::spirit::qi::rule  < Iterator, printable(var\_origin),   whitespace\_grammar< Iterator > > | printable\_r |
|  | |
| boost::spirit::qi::rule  < Iterator, std::string(),   whitespace\_grammar< Iterator > > | printable\_string\_r |
|  | |
| boost::spirit::qi::rule  < Iterator, std::string(),   whitespace\_grammar< Iterator > > | identifier\_r |
|  | |
| boost::spirit::qi::rule  < Iterator, std::vector  < var\_decl >  ), whitespace\_grammar  < Iterator > > | local\_var\_decls\_r |
|  | |
| boost::spirit::qi::rule  < Iterator, no\_op\_statement(),   whitespace\_grammar< Iterator > > | no\_op\_statement\_r |
|  | |
| boost::spirit::qi::rule  < Iterator, std::vector  < expression >var\_origin),   whitespace\_grammar< Iterator > > | opt\_dims\_r |
|  | |
| boost::spirit::qi::rule  < Iterator, range(var\_origin),   whitespace\_grammar< Iterator > > | range\_r |
|  | |
| boost::spirit::qi::rule  < Iterator, sample(bool,   var\_origin),   whitespace\_grammar< Iterator > > | sample\_r |
|  | |
| boost::spirit::qi::rule  < Iterator, statement(bool,   var\_origin, bool),   whitespace\_grammar< Iterator > > | statement\_r |
|  | |
| boost::spirit::qi::rule  < Iterator,   boost::spirit::qi::locals  < std::vector< var\_decl >  >, statements(bool,   var\_origin, bool),   whitespace\_grammar< Iterator > > | statement\_seq\_r |
|  | |
| boost::spirit::qi::rule  < Iterator, range(var\_origin),   whitespace\_grammar< Iterator > > | truncation\_range\_r |
|  | |
| boost::spirit::qi::rule  < Iterator, variable\_dims(var\_origin),   whitespace\_grammar< Iterator > > | var\_lhs\_r |
|  | |

## Detailed Description

### template<typename Iterator> struct stan::gm::statement\_grammar< Iterator >

Definition at line 19 of file statement\_2\_grammar.hpp.

## Constructor & Destructor Documentation

template<typename Iterator >

|  |  |  |  |
| --- | --- | --- | --- |
| stan::gm::statement\_grammar< Iterator >::statement\_grammar | ( | variable\_map & | *var\_map*, |
|  |  | std::stringstream & | *error\_msgs* |
|  | ) |  |  |

Definition at line 527 of file statement\_grammar\_def.hpp.

## Member Data Documentation

template<typename Iterator>

boost::spirit::qi::rule<Iterator, assignment(var\_origin), whitespace\_grammar<Iterator> > stan::gm::statement\_grammar< Iterator >::assignment\_r

Definition at line 45 of file statement\_grammar.hpp.

template<typename Iterator>

boost::spirit::qi::rule<Iterator, std::vector<expression>var\_origin), whitespace\_grammar<Iterator> > stan::gm::statement\_grammar< Iterator >::dims\_r

Definition at line 56 of file statement\_grammar.hpp.

template<typename Iterator>

boost::spirit::qi::rule<Iterator, distribution(var\_origin), whitespace\_grammar<Iterator> > stan::gm::statement\_grammar< Iterator >::distribution\_r

Definition at line 62 of file statement\_grammar.hpp.

template<typename Iterator>

std::stringstream& stan::gm::statement\_grammar< Iterator >::error\_msgs\_

Definition at line 34 of file statement\_grammar.hpp.

template<typename Iterator>

expression\_grammar<Iterator> stan::gm::statement\_grammar< Iterator >::expression\_g

Definition at line 37 of file statement\_grammar.hpp.

template<typename Iterator>

boost::spirit::qi::rule<Iterator, boost::spirit::qi::locals<std::string>, for\_statement(bool,var\_origin,bool), whitespace\_grammar<Iterator> > stan::gm::statement\_grammar< Iterator >::for\_statement\_r

Definition at line 74 of file statement\_grammar.hpp.

template<typename Iterator>

boost::spirit::qi::rule<Iterator, std::string(), whitespace\_grammar<Iterator> > stan::gm::statement\_grammar< Iterator >::identifier\_r

Definition at line 121 of file statement\_grammar.hpp.

template<typename Iterator>

boost::spirit::qi::rule<Iterator, increment\_log\_prob\_statement(bool,var\_origin), whitespace\_grammar<Iterator> > stan::gm::statement\_grammar< Iterator >::increment\_log\_prob\_statement\_r

Definition at line 68 of file statement\_grammar.hpp.

template<typename Iterator>

boost::spirit::qi::rule<Iterator, std::vector<var\_decl>), whitespace\_grammar<Iterator> > stan::gm::statement\_grammar< Iterator >::local\_var\_decls\_r

Definition at line 126 of file statement\_grammar.hpp.

template<typename Iterator>

boost::spirit::qi::rule<Iterator, no\_op\_statement(), whitespace\_grammar<Iterator> > stan::gm::statement\_grammar< Iterator >::no\_op\_statement\_r

Definition at line 131 of file statement\_grammar.hpp.

template<typename Iterator>

boost::spirit::qi::rule<Iterator, expression(var\_origin), whitespace\_grammar<Iterator> > stan::gm::statement\_grammar< Iterator >::non\_lvalue\_assign\_r

Definition at line 50 of file statement\_grammar.hpp.

template<typename Iterator>

boost::spirit::qi::rule<Iterator, std::vector<expression>var\_origin), whitespace\_grammar<Iterator> > stan::gm::statement\_grammar< Iterator >::opt\_dims\_r

Definition at line 136 of file statement\_grammar.hpp.

template<typename Iterator>

boost::spirit::qi::rule<Iterator, print\_statement(var\_origin), whitespace\_grammar<Iterator> > stan::gm::statement\_grammar< Iterator >::print\_statement\_r

Definition at line 86 of file statement\_grammar.hpp.

template<typename Iterator>

boost::spirit::qi::rule<Iterator, printable(var\_origin), whitespace\_grammar<Iterator> > stan::gm::statement\_grammar< Iterator >::printable\_r

Definition at line 110 of file statement\_grammar.hpp.

template<typename Iterator>

boost::spirit::qi::rule<Iterator, std::string(), whitespace\_grammar<Iterator> > stan::gm::statement\_grammar< Iterator >::printable\_string\_r

Definition at line 115 of file statement\_grammar.hpp.

template<typename Iterator>

boost::spirit::qi::rule<Iterator, range(var\_origin), whitespace\_grammar<Iterator> > stan::gm::statement\_grammar< Iterator >::range\_r

Definition at line 141 of file statement\_grammar.hpp.

template<typename Iterator>

boost::spirit::qi::rule<Iterator, reject\_statement(var\_origin), whitespace\_grammar<Iterator> > stan::gm::statement\_grammar< Iterator >::reject\_statement\_r

Definition at line 91 of file statement\_grammar.hpp.

template<typename Iterator>

boost::spirit::qi::rule<Iterator, return\_statement(var\_origin), whitespace\_grammar<Iterator> > stan::gm::statement\_grammar< Iterator >::return\_statement\_r

Definition at line 97 of file statement\_grammar.hpp.

template<typename Iterator>

boost::spirit::qi::rule<Iterator, sample(bool,var\_origin), whitespace\_grammar<Iterator> > stan::gm::statement\_grammar< Iterator >::sample\_r

Definition at line 146 of file statement\_grammar.hpp.

template<typename Iterator>

statement\_2\_grammar<Iterator> stan::gm::statement\_grammar< Iterator >::statement\_2\_g

Definition at line 39 of file statement\_grammar.hpp.

template<typename Iterator>

boost::spirit::qi::rule<Iterator, statement(bool,var\_origin,bool), whitespace\_grammar<Iterator> > stan::gm::statement\_grammar< Iterator >::statement\_r

Definition at line 151 of file statement\_grammar.hpp.

template<typename Iterator>

boost::spirit::qi::rule<Iterator, boost::spirit::qi::locals<std::vector<var\_decl> >, statements(bool,var\_origin,bool), whitespace\_grammar<Iterator> > stan::gm::statement\_grammar< Iterator >::statement\_seq\_r

Definition at line 157 of file statement\_grammar.hpp.

template<typename Iterator>

boost::spirit::qi::rule<Iterator, range(var\_origin), whitespace\_grammar<Iterator> > stan::gm::statement\_grammar< Iterator >::truncation\_range\_r

Definition at line 162 of file statement\_grammar.hpp.

template<typename Iterator>

var\_decls\_grammar<Iterator> stan::gm::statement\_grammar< Iterator >::var\_decls\_g

Definition at line 38 of file statement\_grammar.hpp.

template<typename Iterator>

boost::spirit::qi::rule<Iterator, variable\_dims(var\_origin), whitespace\_grammar<Iterator> > stan::gm::statement\_grammar< Iterator >::var\_lhs\_r

Definition at line 167 of file statement\_grammar.hpp.

template<typename Iterator>

variable\_map& stan::gm::statement\_grammar< Iterator >::var\_map\_

Definition at line 33 of file statement\_grammar.hpp.

template<typename Iterator>

boost::spirit::qi::rule<Iterator, return\_statement(var\_origin), whitespace\_grammar<Iterator> > stan::gm::statement\_grammar< Iterator >::void\_return\_statement\_r

Definition at line 102 of file statement\_grammar.hpp.

template<typename Iterator>

boost::spirit::qi::rule<Iterator, while\_statement(bool,var\_origin,bool), whitespace\_grammar<Iterator> > stan::gm::statement\_grammar< Iterator >::while\_statement\_r

Definition at line 80 of file statement\_grammar.hpp.

---

The documentation for this struct was generated from the following files:

- src/stan/gm/grammars/statement\_2\_grammar.hpp
- src/stan/gm/grammars/statement\_grammar.hpp
- src/stan/gm/grammars/statement\_grammar\_def.hpp


---

[ Stan Home Page ]

*© 2011–2014,
Stan Development Team.*
